# Background sequence characteristics influence the occurrence and severity of disease-causing mtDNA mutations

**DOI:** 10.1371/journal.pgen.1007126

**Published:** 2017-12-18

**Authors:** Wei Wei, Aurora Gomez-Duran, Gavin Hudson, Patrick F. Chinnery

**Affiliations:** 1 MRC-Mitochondrial Biology Unit, Cambridge Biomedical Campus, Cambridge, United Kingdom; 2 Department of Clinical Neurosciences, Cambridge Biomedical Campus, University of Cambridge, Cambridge, United Kingdom; 3 Institute of Genetic Medicine, Central Parkway, Newcastle University, Newcastle upon Tyne, United Kingdom; Max Planck Institute for Biology of Ageing, GERMANY

## Abstract

Inherited mitochondrial DNA (mtDNA) mutations have emerged as a common cause of human disease, with mutations occurring multiple times in the world population. The clinical presentation of three pathogenic mtDNA mutations is strongly associated with a background mtDNA haplogroup, but it is not clear whether this is limited to a handful of examples or is a more general phenomenon. To address this, we determined the characteristics of 30,506 mtDNA sequences sampled globally. After performing several quality control steps, we ascribed an established pathogenicity score to the major alleles for each sequence. The mean pathogenicity score for known disease-causing mutations was significantly different between mtDNA macro-haplogroups. Several mutations were observed across all haplogroup backgrounds, whereas others were only observed on specific clades. In some instances this reflected a founder effect, but in others, the mutation recurred but only within the same phylogenetic cluster. Sequence diversity estimates showed that disease-causing mutations were more frequent on young sequences, and genomes with two or more disease-causing mutations were more common than expected by chance. These findings implicate the mtDNA background more generally in recurrent mutation events that have been purified through natural selection in older populations. This provides an explanation for the low frequency of mtDNA disease reported in specific ethnic groups.

## Author summary

MtDNA mutations are a major cause of genetic disease. Many of these variants have recurred several times in different populations and on diverse haplogroup backgrounds, but the clinical presentation of mutations causing Leber Hereditary Optic Neuropathy (LHON: m.14484T>C, m.3460G>A, m.11778G>A) are strongly associated with a specific mtDNA haplogroup. This raises the possibility that many pathogenic mtDNA mutations are subject to the same effects. Here, our analysis of 30,506 human mtDNA sequences shows that the association between disease-causing mtDNA mutations and background mtDNA haplogroups is not only restricted to three disease-causing mtDNA mutations known to cause LHON. The frequent recurrence of the same mutations on a population clade, and the reduced frequency of European mtDNAs harboring two or more diseases-causing mutations, suggest that the population mtDNA background influences the risk of developing mtDNA mutations. Our analysis also shows that disease-causing mtDNA mutations also occur more frequently on younger mtDNAs. This implies that, once formed, the mutations are selected against. These findings indicate that the clinical interpretation of mtDNA variants should be performed within an ethnogeographic context.

## Introduction

Human mitochondrial DNA (mtDNA) is exclusively maternally inherited and undergoes negligible recombination at the population level. As humans emerged from Africa and populated the globe, different sub-populations acquired single nucleotide variants (SNVs) that define geographically-restricted mtDNA ‘haplogroups’[1]. Some rare genetic variants have emerged as a common cause of inherited metabolic disease, affecting 1 in 10,000 of the population[2]. Many of these variants have recurred several times in different populations and on diverse haplogroup backgrounds, but the clinical presentation of mutations causing Leber Hereditary Optic Neuropathy (LHON: m.14484T>C, m.3460G>A, m.11778G>A) are strongly associated with a specific mtDNA haplogroup[3, 4]. This raises the possibility that many (but not all[5]) pathogenic mtDNA mutations are subject to the same effects. This has not been systematically studied to date, in large part because of the limited number of sequences available for analysis.

## Results

### Data description and quality control

**Fig 1:**
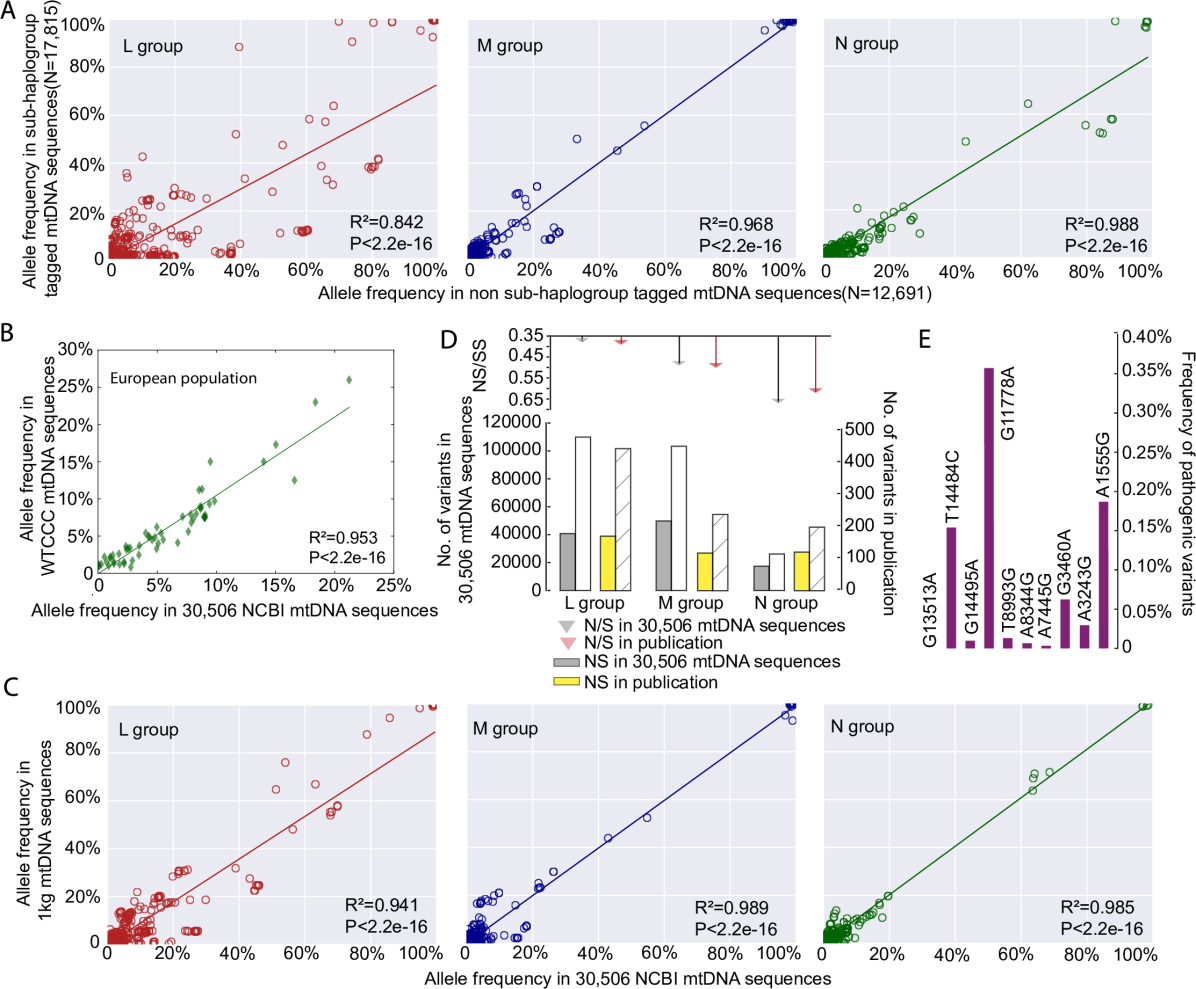
mtDNA sequence quality control. (A) Correlation of allele frequencies of the variants between 17,815 sub-haplogroup tagged mtDNA sequences and the remaining 12,691 non sub-haplogroup tagged mtDNA sequences. (B) Correlation of 59 mtDNA variant frequencies between 9,935 independent population controls from the Wellcome Trust Case Control Consortium and this study. (C) Correlation of allele frequencies of the variants between 1,370 mtDNA sequences from 1000 Genome Project (1kg) and 30,506 GenBank NCBI mtDNA sequences. (D) Ratio of non-synonymous (NS) to synonymous (S) mtDNA variants in 30,506 mtDNA sequences compared to an independent published dataset [9], the numbers of NS and S are shown in bar chart at the bottom. (E) Allele frequencies of ten common disease-causing mutations. There was no difference when compared to previously published values determined through a population-based study in sequential healthy live-births in Europeans[10].

30,506 mtDNA sequences were downloaded from GenBank (**S1 Table and S2 Table**). These included 3,852 sequences from macro-haplogroups L, 6,202 from M and 20,452 from N. In 17,815 of the 30,506 mtDNA sequences it was possible to identify all known appropriate haplogroup markers down to the sub-haplogroup level [6, 7] (http://www.phylotree.org/tree/index.htm) (referred to as ‘sub-haplogroup tagged’ **S1 Table**). Next we compared the frequency of the variants to the remaining 12,691 sequences (referred to as non-sub-haplogroup tagged), stratifying for each macro-haplogroup (2,710 sequences from L, 2,619 from M, and 7,362 from N; **S1 Table**). The allele frequencies were strongly correlated between the two groups (R^2^ = 0.842, *p*-value < 2.2e-16 for macro-haplogroup L; R^2^ = 0.968, *p*-value < 2.2e-16 for macro-haplogroup M; R^2^ = 0.988, *p*-value < 2.2e-16 for macro-haplogroup N, Person’s correlation test; **Fig 1A, S1A Fig**).

Next we compared the allele frequencies in the 17,790 European haplogroup N(R) sequences to an independent sample of 9935 population controls from the Wellcome Trust Case Control Consortium [8]. Allele frequencies from the 59 mtDNA variants present in both datasets were highly correlated (R^2^ = 0.953, *p*-value < 2.2e-16, Person’s correlation test; **Fig 1B**). We then compared 30,506 mtDNA sequences with 1,370 mtDNA sequences from 1000 Genome Project (see Methods), which includes mtDNA sequences from across the world (462 from macro-haplogroup L, 271 from M, and 637 from N). The allele frequencies of the mtDNA variants were highly correlated between two datasets for each macro-haplogroup (R^2^ = 0.941, *p*-value < 2.2e-16 for macro-haplogroup L; R^2^ = 0.989, *p*-value < 2.2e-16 for macro-haplogroup M; R^2^ = 0.985, *p*-value < 2.2e-16 for macro-haplogroup N; Pearson’s correlation test; **Fig 1C, S2 Fig, S3 Fig**). Further evidence of quality control came from an independent comparison of the GenBank dataset to 1,125 published global human mtDNA sequences from the literature [9], where the ratios of non-synonymous variants (NS) and synonymous variants (SS) within major haplogroups were no different (*p*-value > 0.05, Fisher test; **Fig 1D**). Finally the allele frequencies of common disease-causing mutations were no different to previously published values derived from a healthy population birth cohort in Europeans (i.e. an established ‘population carrier frequency’ for these mutations, *p*-value > 0.05, Fisher test; **Fig 1E**)[10], providing further evidence that our dataset is not significantly enriched with sequences derived from patients with inherited mtDNA diseases in the European population. A similar comparison was not currently possible for African and Asian mtDNAs. However, we saw no relationship the pathogenicity score of specific alleles and the difference in allele frequency and sub-haplogroup tagged *vs*. non-sub-haplogroup tagged mtDNAs in our data set (See Methods for an explanation of pathogenicity scores, **S1B Fig**). When taken together, these analysis (**Fig 1A–1E**), indicate that the 30,506 GenBank sequences are a representative sample of the background populations and not significantly enriched for rare population variants or pathogenic mutations identified through patients presenting with mitochondrial diseases.

### Determining the disease caused mutations

**Fig 2:**
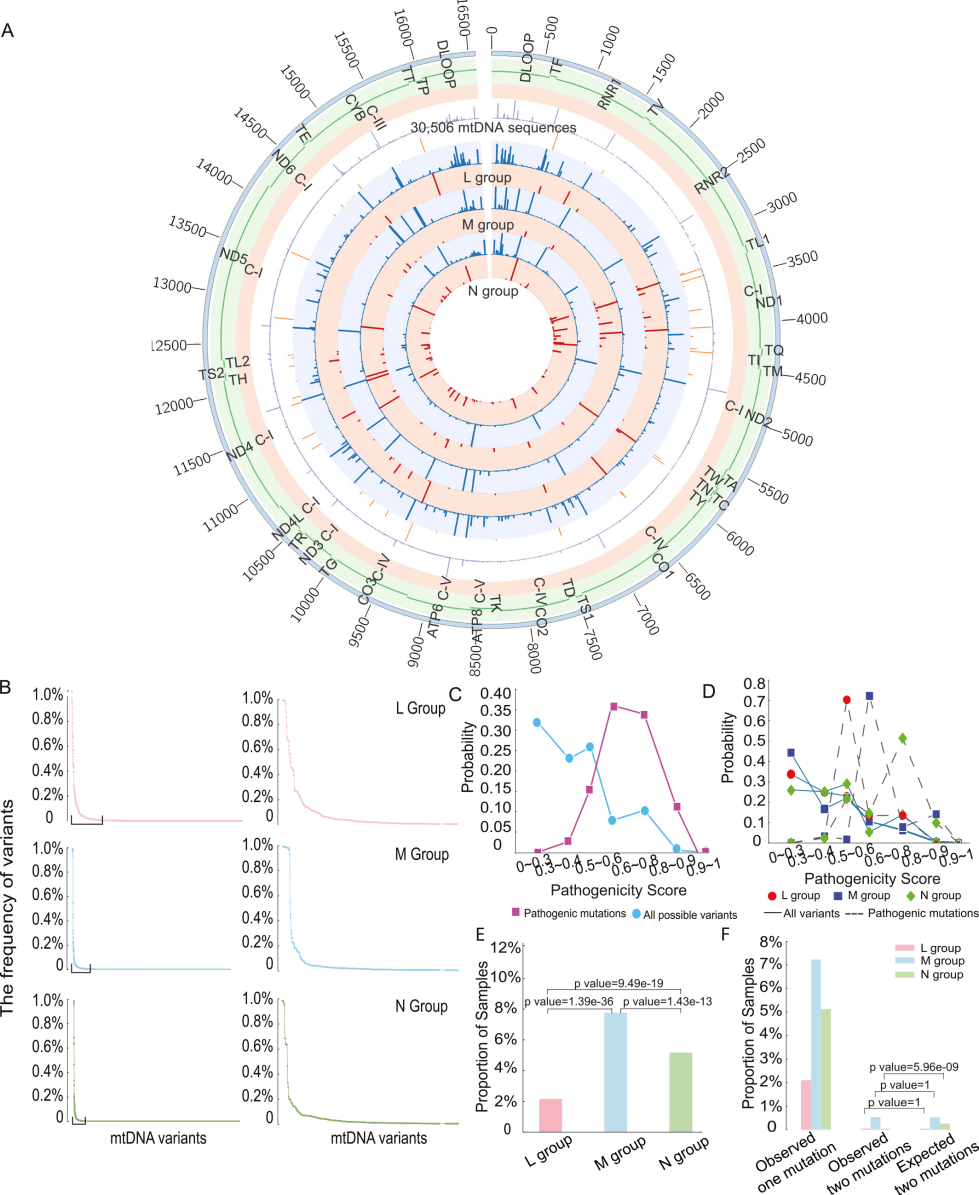
The distribution of variant frequency and assessing the pathogenicity score in 30,506 mtDNA sequences. (A) Circos plot summarizing all of the genetic data in 30,506 mtDNA sequences. From outside the circle to inside: (1) mtDNA position, (2) mtDNA genes, (3) mtDNA Complex, (4) frequency of all variants in 30,506 mitochondrial sequences(range 0 to 98.70%), (5) frequency of diseases-causing mutations in 30,506 mitochondrial sequences(range 0 to 0.89%), (6) frequency of all variants in L group(range 0 to 99.45%), (7) frequency of diseases-causing mutations in L group(range 0 to 0.65%), (8) frequency of all variants in M group(range 0 to 99.60%), (9) frequency of diseases-causing mutations in M group(range 0 to 3.11%), (10) frequency of all variants in N group(range 0 to 98.60%), (11) frequency of diseases-causing mutations in N(range 0 to 1.26%), Color code for circles (4)–(11): Red—frequency of diseases-causing mutations, blue—frequency of all variants. (B) The distribution of frequency of variants in each macro-haplogroup. MtDNA variants in were ordered based on frequency from high to low. The right-hand panel highlights the variants with frequency above 0.5% in each group. (C) Probability distributions of the observed pathogenicity scores for all population variants and defined disease-causing mutations. (D) Probability distributions of the pathogenicity scores for all variants and disease-causing mutations within each macro-haplogroup. (E) Proportion of samples carrying disease-causing mutations. (F) Percentage of mtDNA sequences harboring two of more disease-causing mutations.

The 30,506 mtDNA sequences were predicted to cause 10,166 unique single amino acid substitutions (L = 3,732, M = 4,887, N = 8,427, **Fig 2A**). There was no difference in the frequency distribution for mtDNA variants between the macro-haplogroups (*p*-value = 1, ANOVA test; **Fig 2B**). Next we determined which variants were likely to be pathogenic. Ninety of the 202 proposed pathogenic mtDNA variants listed on the ClinVar database[11] were found in the 30,506 mtDNA sequences studied here (**S3 Table**). Incorporating published and on-line evidence of pathogenicity (see Methods), the number of likely pathogenic mutations reduced from 90 to 57(**S4 Table**).

### Pathogenicity of variants in macro-haplogroups L, M and N

We initially focused on the pathogenicity of all variants within protein-encoding genes. 2,709 variants were present in 30,506 sequences, including 34 disease-causing mutations (**S4 Table**). As expected, variants with a higher pathogenicity score were less frequent in the population, and disease-causing mutations with a higher pathogenicity score were less frequent in the population (**Fig 2C**). Likewise, as expected, the probability distribution of pathogenicity scores for disease-causing mutations was significantly greater than the overall distribution of pathogenicity scores in the entire population (mean = 0.59 for disease-causing mutations, mean = 0.37 for all possible variants; two-sample t-test, *p*-value < 2.2e-16, **Fig 2C**). However, we were surprised to see that disease-causing mutations on the macro-haplogroup N and M backgrounds had significantly higher pathogenicity score than those occurring on the macro-haplogroup L background (mean = 0.47 for L group, mean = 0.62 for M group, mean = 0.59 for N group; L versus M: *p*-value = 1.959e-11, L versus N: *p*-value = 7.992e-08, M versus N: *p*-value = 8.967e-07, two-sample t test, **Fig 2D**). These differences could not be accounted for by the frequency of specific mutations, which did not follow the same trend (**S4 Fig**). 83(2.2%) mtDNA sequences carried at least one disease-causing mutation in L group, 481 (7.8%) in M group and 1,057 (5.2%) in N group. Group M sequences had a higher proportion of sequences carrying disease-causing mutations 481 (7.8%), followed by group N sequences 1,057 (5.2%), with the lowest in group L 83 (2.2%) (L versus M: *p*-value = 1.39e-36, L versus N: *p*-value = 9.49e-19, M versus N: *p*-value = 1.43e-13, Fisher test, **Fig 2E**).

Based on the overall frequency of disease causing mutations, we estimated the likelihood of a second mutation occurring by chance on the same genome (**Fig 2F**). For macro-haplogroups L and M, the observed frequency of mtDNAs with two of more disease-causing mutations was no different to the chance co-occurrence. However, for macro-haplogroup N sequences, the number of mtDNA sequences harboring two of more disease-causing mutations was 6-fold lower than expected by chance (*p*-value = 5.96e-09, Fisher test; **Fig 2F**). These observations implicate the mtDNA sequence background in the likelihood of acquiring or retaining a disease-causing mutation.

**Fig 3:**
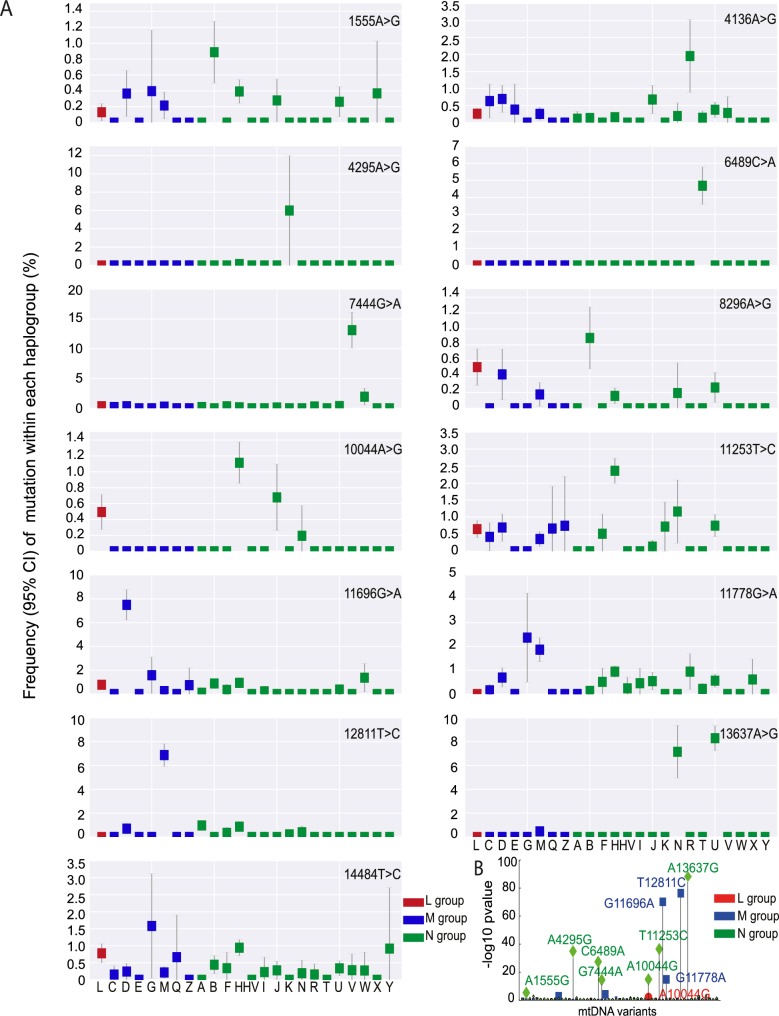
Frequency of disease causing mtDNA mutations in each macro-haplogroup. (A) Frequency of 13 disease causing mutations present at >0.1% frequency in each haplogroup. (B) Disease-causing mutations significantly associated with specific mtDNA haplogroups. Uncorrected p-value thresholds are shown.

We then determined the frequency of the 57 disease-causing mutations on the ‘major’ haplogroups. Twenty-five (43.9%) of the 57 diseases-causing mutations were present on more than one macro-haplogroup, and 34 (59.6%) were present on more than one major haplogroup (**Fig 3A, S5 Fig**), indicating recurrent mutation events. 23 (40.4%) of the pathogenic mutations were only found on one haplogroup. Of these, m.4136A>G, m.4317A>G, m.7444G>A, m.11696G>A. and m.13637A>G were recurrent mutations based on analysis of the entire mtDNA sequence. This suggests that specific mtDNA mutations recur on specific mtDNA haplogroups, implying that the background haplogroup predisposes to the original mutation event, or the subsequent fixation of the allele on a particular maternal lineage. On the other hand, m.5631G>A (L), m.10663T>C (L), m.8313G>A (M), m.12770A>G (M), and m.3890G>A (N) were only seen on one clade, consistent with a single founder event. This raises the possibility that these several ‘disease-causing’ mutations are actually rare sub-haplogroup markers and not directly pathogenic (eg m.4295A>G, m.6489C>A). Overall, we found statistical evidence of haplogroup clustering for 10 (29.4%) of the 34 recurrent disease-causing mtDNA mutations after correcting for the sample size within each comparison (*p*-value < 0.01, Fisher test; **Fig 3B**).

### Mutational signature

**Fig 4:**
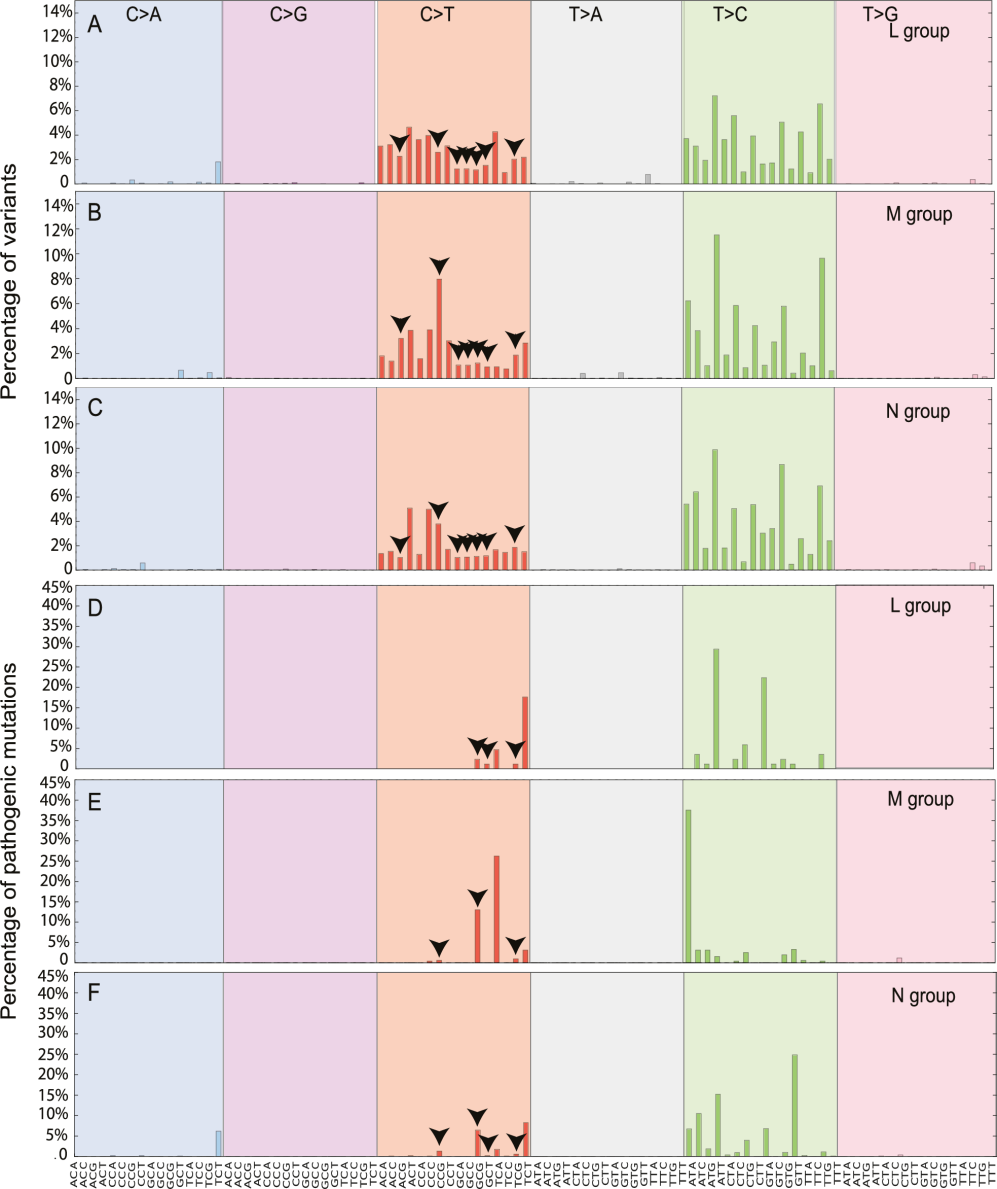
The mutational signatures observed in 30,506 mtDNA sequences. Each signature is displayed according to the 96 substitution classification defined by the substitution class and sequence context immediately 3’ and 5’ to the mutated base. The probability bars for the six types of substitutions are displayed in different colors and labeled at the top of the graph. The mutation types are on the horizontal axes at the bottom of the graph. (A) All possible variants in macro-haplogroup L. (B) All possible variants in macro-haplogroup M. (C) All possible variants in macro-haplogroup N. (D) All diseases-causing mutations in macro-haplogroup L. (E) All diseases-causing mutations in macro-haplogroup M. (F) All diseases-causing mutations in macro-haplogroup N. The arrows highlight the variants in CpG dinucleotides.

We then determined the relative frequency of mutations within all 96 possible flanking nucleotide triplets[12]. Overall, across all macro-haplogroups we saw a greater prominence of C>T/A>G and T>C/A>G substitutions for all variants (p< 2.2e-16, Fisher test; **Fig 4A–4C**), and for disease causing mutations (p< 2.2e-16, Fisher test; **Fig 4D–4F**). In order to confirm the prominence of C>T and T>C mutations observed were not due to the selection of the rCRS [13] as the “reference” by which the mutations are classified, we realigned 30,506 mtDNA sequences to Reconstructed Sapiens Reference Sequence (RSRS) [14]. We observed similar patterns, with T>C and C>T substitutions frequently observed (**S6 Fig**), suggesting the profiles of mutational signatures were not driven by the alignment process and are independent of the reference sequence used in the analysis.

We also observed that the higher mutability of CpG dinucleotides in disease-causing mutations when compared to all variants (CpG%, *p*-value = 0.00017, Fisher exact test; **S7 Fig and S5 Table**). Although the methylation of mtDNA appears to be rare in somatic tissues [15], nuclear genome methylation changes substantially during embryonic and germ-line development [16], raising the possibility that methylation of mtDNA CpGs during early development contributes to the origin of *de novo* mtDNA mutations [17]. Intriguingly, macro-haplogroup M had highest CpG% for all variants (19.93%; L versus M, *p*-value < 2.2e-16 and M versus N *p*-value < 2.2e-16 Fisher exact test) and defined diseases-causing mutations (21.21%; L versus M, *p*-value = 0.00049; and M versus N, *p*-value = 1.06e-06). By contrast, macro-haplogroup L had much lower CpG% than other two groups for disease-causing mutations (5.88%; L versus M *p*-value = 0.00049 and L versus N *p*-value = 0.15) (**S7 Fig and S6 Table**). These findings add further weight to the hypothesis that haplogroup-specific sequence context influences the acquisition of mtDNA mutations.

### The age of mtDNA sequences

**Fig 5:**
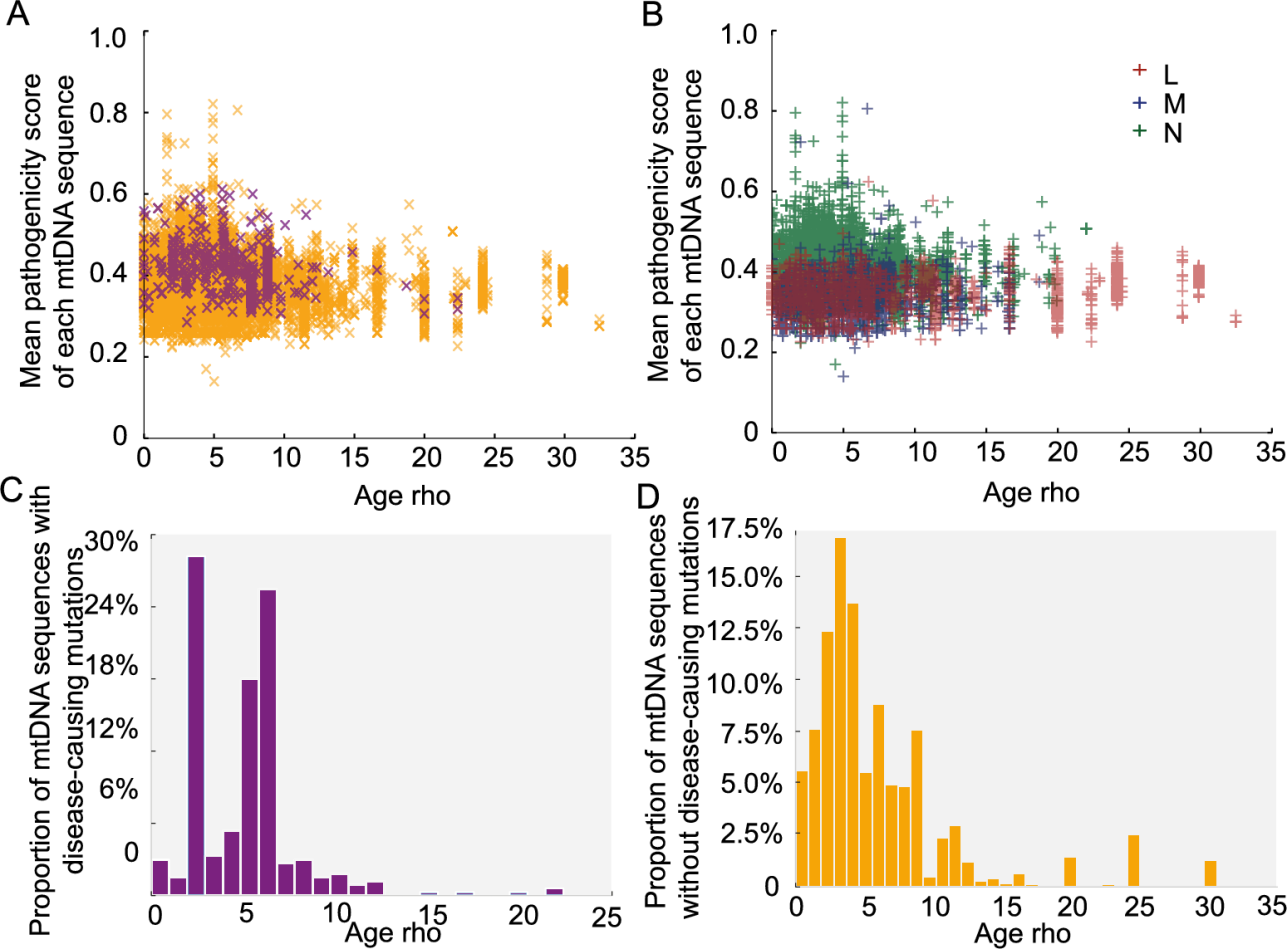
Predicted age of each mtDNA sequence. Age calculations are based on the Rho. (A) Distribution of the mean pathogenicity scores of each mtDNA sequence and predicted age (Rho). Orange “x”: mtDNA sequence not carrying disease-causing mutations; Purple “x”: mtDNA sequence carrying disease-causing mutations. (B) Distribution of mean pathogenicity scores of each mtDNA sequence and predicted age (Rho) (C) Distribution of mtDNA sequences with disease-causing mutations according to predicted age (Rho). (D) Distribution of mtDNA sequences with non-disease-causing mutations according to predicted age (Rho).

We then estimated the age of the each mtDNA sequence based on the major haplogroup classification, using the Rho statistic[18]. Overall, variants with higher pathogenicity scores were found on ‘younger’ (more recent) mtDNA sequences (**Fig 5A and 5B,** *p*-value < 2.2e-16, Wilcoxon Rank test). Disease-causing mutations were also found on younger mtDNA sequences (**Fig 5A**, *p*-value = 0.019, Wilcoxon Rank test). When we studied the same relationship after removing the main pathogenic allele from each sequence *in silico* (**Fig 5C and 5D**), we did not observe an association between the remaining sequence and the age of predicted age of the sequence. This indicates that the association between disease-causing variants and the age of the mitochondrial genome is driven by the pathogenic variant itself and not the entire mtDNA sequence. Although it is possible that Rho dating may be inaccurate in the absence of population expansions [19], these observations are in keeping with the effects of natural section acting on specific pathogenic variants.

### The pathogenicity of mtDNA tRNAs

**Fig 6:**
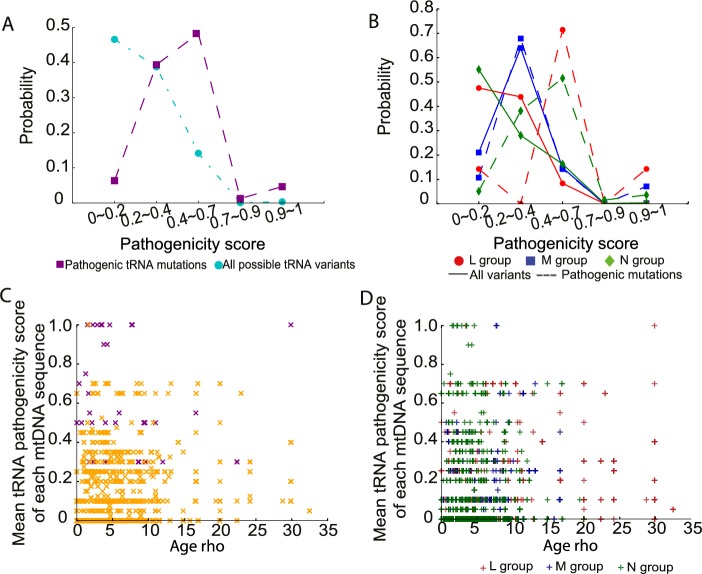
Pathogenicity of mtDNA tRNA variants in 30,506 mtDNA sequences. (A) Probability distributions of the observed pathogenicity scores for all tRNA variants and defined disease-causing tRNA mutations. (B) Probability distributions of the pathogenicity scores for all tRNA variants and disease-causing tRNA mutations within each macro-haplogroup. (C) Distribution of the mean pathogenicity scores for tRNA variants of each mtDNA sequence and predicted age (Rho). Orange “x”: mtDNA sequence not carrying disease-causing tRNA mutations; Purple “x”: mtDNA sequence carrying disease-causing tRNA mutations. (D) Distribution of mean pathogenicity scores for tRNA variants of each mtDNA sequence and predicted age (Rho) within each macrohaplogroup.

Finally, we estimated the pathogenicity scores for 207 variants in tRNA genes [20, 21]. Seventy-four tRNA variants were present in 30,506 sequences, including 16 previously defined disease-causing mutations (**S4 Table**). Consistent with the pathogenicity scores for non-synonymous variants, all tRNA variants and disease-causing tRNA mutations with a higher pathogenicity score were less frequent in the population (**Fig 6A**). Likewise, the probability distributions for disease-causing tRNA mutations was significantly greater than the overall distribution of pathogenicity scores in the entire population (mean = 0.427 for disease-causing tRNA mutations, mean = 0.102 for all possible tRNA variants; *p*-value < 2.2e-16, two-sample t test; **Fig 6A**). Again, we observed that disease-causing tRNA mutations on macro-haplogroups N and M had significantly higher pathogenicity score than those occurring on macro-haplogroup L (mean = 0.36 for L group, mean = 0.51 for M group, mean = 0.43 for N group; L versus M: *p*-value = 0.010, L versus N: *p*-value = 0.005, M versus N: *p*-value = 0.079, two- sample t test; **Fig 6B**). However, there was no correlation between either all tRNA variants or disease-causing tRNA mutations with the ages of the mtDNA sequences (**Fig 6C and 6D**). This could reflect the limited number of tRNA variants in the data set, or that the effects of selection cannot be detected for homoplasmic tRNA gene variants. This is in keeping with animal data, where there is severe selection against protein coding variants within one or two generations, but not tRNA variants[22].

## Discussion

Our analysis of 30,506 human mtDNA sequences shows that the association between disease-causing mtDNA mutations and background mtDNA haplogroups is not only restricted to three disease-causing mtDNA mutations known to cause LHON. The frequent recurrence of the same mutations on a population clade, and the reduced frequency of European mtDNAs harboring two or more diseases-causing mutation, both suggest that the population mtDNA background influences the risk of developing mtDNA mutagenesis. Although it is conceivable that this is due to the functional consequences of haplogroup variants, perhaps though the generation of increased oxygen free radicals, or the segregation of heteroplasmic variants, our analysis indicates that this is unlikely because we also saw an association of mutations with underlying sequence characteristics (such as CpG%) that are not directly linked to oxidative phosphorylation or heteroplasmy segregation.

Our analysis also shows that disease-causing mtDNA mutations also occur more frequently on younger mtDNAs. This implies that, once formed, the mutations are selected against. Although tempting to think that this pattern simply reflects an ascertainment bias related to the number of defined disease-causing mtDNA mutations in populations of European origin, the data suggests that this is not the case. Firstly, the majority of mtDNA mutations were not restricted to specific continental populations; and secondly a greater proportion of known disease-causing mutations were seen in the sequences derived from the Asian population (**Fig 3 and S5 Fig**). To our mind, the simplest explanation is that the more severe homoplasmic mutations have been removed from the older populations through natural selection. A similar pattern has been noted previously in older African mtDNA sequences[23], which have been exposed to the effects of purifying selection for a longer period of time. This further endorses the validity of our dataset and analysis. Thus, both a predilection to mutate and natural selection explain the associations between different mtDNA haplogroups and known-pathogenic mutations, thereby explaining why different ethnic groups are more or less likely to present with a specific mtDNA disease. However, it is important to note that other population genetic effects could have influenced the current mtDNA landscape. For example, recent ancient mtDNA studies point to a European population bottleneck ~14,500 years ago[24], which could also explain the relative lack of evidence of selection in extant Europeans.

Our study was restricted to the major alleles within each mtDNA sequence, and not designed to study the effects of mtDNA heteroplasmy. The prospective analysis of mtDNA heteroplasmy and the segregation of heteroplasmic variants in the population adds a further complexity, but might cast further light on the mechanisms we propose. However, we did observe common heteroplasmic mtDNAs within the dataset across all populations (eg. m.3243A>G), and these variants followed the same overall patterns described here. In addition, no phenotypic data was available. Although a limitation, even without this information we can draw some conclusions. First, despite the well-established strong association between LHON families harboring m.3460G>A, m.11778G>A and m.14484T>C and mtDNA haplgroup J, at the population level, these variants are found across a broad range of mtDNA haplogroups in all three continental groups (**Fig 3**). In this study, m.14484T>C was found on multiple haplogroups, with an allele frequency up to 1.5% on haplogroup M(G). This contrasts sharply with the almost exclusive association of m.14484T>C with haplogroup J described in epidemiological studies of LHON[3, 4]. This strongly supports the view that haplogroup J modifies the clinical penetrance of the m.3460G>A, m.11778G>A, and m.14484T>C mutations, and that the association is not due to an increased mutation rate. Although we cannot be certain, this indicates that the mutation has a reduced penetrance in certain contexts, perhaps related to the nuclear genetic background or environmental factors. Whichever is the case, our findings indicate that the clinical interpretation of mtDNA variants should be performed within an ethnogeographic context.

## Materials and methods

### mtDNA sequence source

30,619 human mtDNA sequences sampled from across the globe downloaded from GenBank (https://www.ncbi.nlm.nih.gov/genbank/). 113 were removed due to poor sequence quality. 30,506 sequences were included in this study and aligned to Revised Cambridge Reference Sequence (rCRS)[13] and Reconstructed Sapiens Reference Sequence (RSRS)[14] using BLASTn (**S1 Table**).

### Phylogenetic analysis

Haplogroup assignment was performed using HaploGrep 2[7]. Sequences were classified into macro-haplogroups (L, M and N), major and sub-haplogroups based on the mtDNA phylogenetic tree[6] (http://www.phylotree.org/tree/index.htm) **(S2 Table),** including 3,852, 6,202 samples and 20,452 samples, respectively. Coalescence times were estimated using the ρ statistic (average distance of the haplotypes of a clade from the respective root haplotype) accompanied by a heuristic estimate of the standard error (σ) calculated from an estimate of the genealogy[18].

### Sequence quality control datasets

#### ‘Sub-haplogroup tagged’ mtDNA sequences in GenBank database

We selected the ‘sub-haplogroup tagged’ mtDNA sequences it was possible to identify all known appropriate haplogroup markers down to the sub-haplogroup level [6, 7] (http://www.phylotree.org/tree/index.htm), including 1,142 sequences from macro-haplogroups L, 3,583 from M and 13,090 from N(**S1 Table**). Next we compared the frequencies of the variants to the remaining 12,691 sequences (non- sub-haplogroup tagged), stratifying for each macro-haplogroup (2,710 sequences from L, 2,619 from M and 7,362 from N; **S1 Table**). The allele frequency (AF) difference of each variant between two groups was calculated as log2 (ratio of frequencies from two groups).

#### mtDNA sequences in 1000 genome project

mtDNA sequences from 2,182 samples were downloaded from 1000 Genome Project (http://www.internationalgenome.org/). mtDNA haplogroup of each sequence was predicted using HaploGrep 2[7] and mt-classifier[21]. We excluded the low quality mtDNA sequences using the following criteria: 1) when <90% of the known haplogroup markers were present for each mtDNA sequence; 2) when more than one haplogroup prediction was reported; 3) when the haplogroups predicted by two prediction tools were inconsistent. There were 1,370 mtDNA sequences, including 462 sequences from macro-haplogroups L, 271 from M and 637 from N for the further QC analysis.

#### Other published mtDNA sequences

We compared the ratio of non-synonymous to synonymous substitutions of 3,852 mtDNA sequences in macro-haplogroup L, 6,202 in M and 2,662 in N (nonR) from this study to 1,125 published global human mtDNA sequences (445 mtDNA sequences in macro-haplogroup L, 239 in M and 199 in N (nonR))[9], across the entire mtDNA phylogenetic tree. For the 17,790 European haplogroup N(R) sequences we also compared the frequency of 59 mtDNA variants to 9,935 population controls from the Wellcome Trust Case Control Consortium[8].

### Determining the disease caused mutations

We determined which variants were likely to be pathogenic. We listed the variants which were present in 30,506 mtDNA sequences and also proposed pathogenic mtDNA variants on the ClinVar database[11]. We did not include data where two or more nucleotide variations and Indels were listed (**S4 Table**). We then reviewed published and on-line evidence of pathogenicity for each one of the 90 variants using the following criteria: (1) Reported more than once with mtDNA disease and/or (2) Documented heteroplasmy, with evidence of segregation with the phenotype at the clinical or biochemical level.

### Pathogenicity measure

We followed the approach first described by Pereira et al. which was based on MutPred software applied to the 13 protein coding genes of mtDNA [25–27]. The details and a list of pathogenicity scores for 24,206 possible amino acid variations are available in Table S3 in Pereira et al. [27]. The pathogenicity scores of variants in tRNA genes were retrieved from the literature [20, 21].

### Mutational signature

Mutational spectra were derived directly from the both rCRS [13] and RSRS mtDNA[14] reference sequences and alternative alleles at each variant site. The resulting spectra are composed of the relative frequencies of the six distinguishable point mutations (C:G>T:A, T:A>C:G, C:G>A:T, C:G>G:C, T:A>A:T and T:A>G:T). Each signature was displayed using a 96 substitution classification defined by the substitution class and the sequence context immediately 3’ and 5’ to the mutated base[12].

### Statistical analysis

The p-values for all comparisons of mean values were calculated by using two-sample t tests or Wilcoxon Rank test as appropriate. Variant counts or fractions were performed using Fisher’s exact test. Pearson’s correlation coefficient (R^2^) and the significance were calculated using the correlation test. All statistical analyses were performed using R (v3.3) (https://www.r-project.org).

### Data availability

The mtDNA sequences that support the findings of this study are available in Genbank: https://www.ncbi.nlm.nih.gov/genbank/. Accession numbers are supplied in S1 Table. No other data is generated specifically in this study.

## Supporting information

S1 Fig Correlation of allele frequency (AFs) of the variants between 17,815 sub-haplogroup tagged mtDNA sequences and 12,691 non sub-haplogroup tagged sequences in NCBI database. (a) The allele frequencies of variants in 12,691 non sub-haplogroup tagged sequences were highly correlated with 17,815 sub-haplogroup tagged sequences within each marco-haplogroup. AFs were shown in log2(AF/1-AF). (b)There was no relationship between the AF difference of sub-haplogroup tagged sequences(AF_tagged) and non sub-haplogroup tagged sequences(AF_non-tagged) with the pathogenic scores of variants in each macro-haplogroup.
(EPS)

S2 Fig Correlation of allele frequency (AFs) of the variants between 1000 Genome Project and 30,506 NCBI mtDNA sequences, AFs were shown in log2(AF/1-AF). The allele frequencies of variants in 1000 Genome Project were highly correlated with 30,506 NCBI mtDNA sequences within each marco-haplogroup. L, M and N groups were shown, respectively.
(EPS)

S3 Fig Correlation of allele frequencies (AFs) of non haplogroup makers between 1000 Genome Project and 30,506 NCBI mtDNA sequences, AFs were shown in log2(AF/1-AF). Macro-haplogroup L, M and N were shown in different colors, respectively.
(EPS)

S4 Fig Correlation of pathogenicity score between polymorphisms and disease-causing mutations. (a) The correlation of mean pathogenicity score between all variants of each mtDNA sequence and only disease-causing mutations of each mtDNA sequence. (b) The correlation of mean pathogenicity score between non-disease-causing mutations of each mtDNA sequence and only disease-causing mutations of each mtDNA sequence.
(EPS)

S5 Fig Comparison of the presence of specific disease causing mtDNA mutations in each macro-haplogroup. Columns left to right: mtDNA variant; mutations specific to macro-haplogroup L (blue); mutations specific to macro-haplogroup M (pink); mutations specific to macro-haplogroup N (green); mutations found on all three macro-haplogroups L,M&N (purple); mutations found on more than one macro-haplogroup (yellow); mutations found on one macro-haplogroup (violet). No = number of variants found in each category.
(EPS)

S6 Fig The categories of mutational signatures observed in 30,506 mtDNA sequences. 30,506 mtDNA sequences were realigned against Reconstructed Sapiens Reference Sequence (RSRS). The probability bars for the six types of substitutions (C>A, C>G, C>T, T>A, T>C and T>G) are displayed within each macro-haplogroup.
(EPS)

S7 Fig Comparison of the distribution of CpG% (%C>T) in macro-haplogroups. (a) Distribution of CpG% (%C>T) in all possible variants. (b) distribution of CpG% (%C>T) in disease-causing mutations.
(EPS)

S1 Table The mtDNA sequences IDs and haplogroup of each sequence used in this study. 17,815 of the 30,506 mtDNA sequences which was possible to identify all known appropriate haplogroup markers down to the sub-haplogroup level were labeled as sub-haplogroup tagged in the QC column.
(XLSX)

S2 Table Sample size of each haplogroup and macro-haplogroup. (DOCX)

S3 Table The details of 202 reported pathogenic variations in ClinVar. (XLSX)

S4 Table The details of 57 defined disease-causing mutations in 30,506 mtDNA sequences. (XLSX)

S5 Table Comparison of the CpG% between all possible variants and disease-causing mutations for the entire population and each group. The percentage of variants in CpG region and the p-value for Fisher’s exact test—all possible variants vs all disease-causing mutations in the entire population and each group were shown and calculated.
(DOCX)

S6 Table Comparison of the CpG% between any two of three groups. The p-value for Fisher’s exact test—each group vs the rest of groups were shown and calculated.
(DOCX)
