# Correction: Background sequence characteristics influence the occurrence and severity of disease-causing mtDNA mutations

**DOI:** 10.1371/journal.pgen.1007364

**Published:** 2018-05-04

**Authors:** Wei Wei, Aurora Gomez-Duran, Gavin Hudson, Patrick F. Chinnery

## Notice of Republication

This article [1] was republished on April 23, 2018, to correct text in the Materials and Methods section, add a citation to the Reference list and remove S7 Table.

The original text under the sub-heading of “Pathogenicity measure” in the Materials and Methods was reproduced from Pereira et al. [2], but not referenced in the article. The republished version has been rewritten to acknowledge the source and add the relevant citation, now included as Reference 27. In addition, S7 Table was originally published as Table S3 in Pereira et al. It was included without attribution and consequently has been removed from the republished article.

All authors agree to these corrections and apologize for the errors.
