# Supplementary material for: Background sequence characteristics influence the occurrence and severity of disease-causing mtDNA mutations
Source: PLoS Genet. 2017 Dec 18;13(12):e1007126. doi: 10.1371/journal.pgen.1007126 (PMC5757940; doi:10.1371/journal.pgen.1007126)
Supplement: S2 Table — (DOCX) [file pgen.1007126.s009.docx]

**S2 Table. Sample size of each haplogroup and macro-haplogroup.**

| **Haplogroup** | **Sample Size** | **Haplogroup** | **Sample Size** |
| --- | --- | --- | --- |
| A | 883 | L6 | 12 |
| B | 2274 | M | 2794 |
| C | 952 | N | 517 |
| D | 1641 | O | 4 |
| E | 262 | P | 84 |
| F | 585 | Q | 165 |
| G | 253 | R | 639 |
| H | 6374 | S | 14 |
| HV | 417 | T | 1410 |
| I | 444 | U | 2666 |
| J | 1477 | V | 480 |
| K | 1384 | W | 365 |
| L0 | 1031 | X | 326 |
| L1 | 707 | Y | 109 |
| L2 | 847 | Z | 135 |
| L3 | 1155 | L - subtree | 3852 |
| L4 | 65 | M - subtree | 6202 |
| L5 | 35 | N - subtree | 20452 |
|  |  | Total Size | 30506 |
