# Supplementary material for: Background sequence characteristics influence the occurrence and severity of disease-causing mtDNA mutations
Source: PLoS Genet. 2017 Dec 18;13(12):e1007126. doi: 10.1371/journal.pgen.1007126 (PMC5757940; doi:10.1371/journal.pgen.1007126)
Supplement: S5 Table — The percentage of variants in CpG region and the p-value for Fisher’s exact test—all possible variants vs all disease-causing mutations in the entire population and each group were shown and calculated. (DOCX) [file pgen.1007126.s012.docx]

**S5 Table. Comparison of the CpG% between all possible variants and disease-causing mutations for the entire population and each group.** The percentage of variants in CpG region and the p-value for Fisher’s exact test - all possible variants vs all disease-causing mutations in the entire population and each group were shown and calculated

| CpG%(%C > T) | All variants | Diseases-causing mutations | p value |
| --- | --- | --- | --- |
| All populations | 15.28%(94.73%) | 19.22%(99.4%) | 0.000171284 |
| L group | 14.20%(93.96%) | 5.88%(100%) | 0.02808074 |
| M group | 19.83%(93.30%) | 21.21%(100%) | 0.438195071 |
| N group | 13.64%(96.25%) | 11.63%(98.91%) | 0.060474625 |
