# Supplementary material for: Background sequence characteristics influence the occurrence and severity of disease-causing mtDNA mutations
Source: PLoS Genet. 2017 Dec 18;13(12):e1007126. doi: 10.1371/journal.pgen.1007126 (PMC5757940; doi:10.1371/journal.pgen.1007126)
Supplement: S6 Table — The p-value for Fisher’s exact test—each group vs the rest of groups were shown and calculated. (DOCX) [file pgen.1007126.s013.docx]

**S6 Table. Comparison of the CpG% between any two of three groups.** The p-value for Fisher’s exact test - each group vs the rest of groups were shown and calculated.

| **Datasets** | **Groups** | **Fisher test p value** |
| --- | --- | --- |
| All variants | L vs M | < 2.2e-16 |
|  | L vs N | 1.56e-11 |
|  | M vs N | < 2.2e-16 |
| Diseases-causing mutations | L vs M | 0.000485635 |
|  | L vs N | 0.149957525 |
|  | M vs N | 1.06e-06 |
